# Effects of COVID-19 Lockdown on Adherence to Individual Home- or Gym-Based Exercise Training among Women with Postmenopausal Osteoporosis

**DOI:** 10.3390/ijerph18052441

**Published:** 2021-03-02

**Authors:** Erika Pinelli, Giuseppe Barone, Sofia Marini, Francesco Benvenuti, Marie H. Murphy, Mikko Julin, Wolfgang Kemmler, Simon Von Stengel, Stefano Di Paolo, Laura Dallolio, Pasqualino Maietta Latessa, Raffaele Zinno, Laura Bragonzoni

**Affiliations:** 1 Department for Life Quality Studies, Campus of Rimini, University of Bologna, Corso d’Augusto 237, 47921 Rimini, Italy; erika.pinelli2@unibo.it (E.P.); giuseppe.barone8@unibo.it (G.B.); benvefrancis@gmail.com (F.B.); stefano.dipaolo4@unibo.it (S.D.P.); pasqualino.maietta@unibo.it (P.M.L.); raffaele.zinno2@unibo.it (R.Z.); laura.bragonzoni4@unibo.it (L.B.); 2 Centre for Exercise Medicine Physical Activity and Health, Ulster University, Newtownabbey, Co Antrim BT37 0QB, UK; mh.murphy@ulster.ac.uk; 3 Department of Physiotherapy, Laurea University of Applied Sciences, 02650 Espoo, Finland; mikko.julin@laurea.fi; 4 Institute of Medical Physics, Friedrich-Alexander-University Erlangen-Nürnberg, 91052 Erlangen, Germany; wolfgang.kemmler@imp.uni-erlangen.de (W.K.); simon.von.stengel@imp.uni-erlangen.de (S.V.S.); 5 Department of Biomedical and Neuromotor Science, University of Bologna, Via San Giacomo 12, 40126 Bologna, Italy; laura.dallolio@unibo.it

**Keywords:** COVID-19, adherence, physical activity, exercise, postmenopausal osteoporosis

## Abstract

Adherence is important for an exercise program’s efficacy. This study aims at investigating whether the COVID-19 lockdown had different consequences on the adherence to an exercise program specifically designed for women with postmenopausal osteoporosis when administered as individual home training (IHT) or gym group training (GGT). At the start of the lockdown, which imposed the temporary closure of any gym activities, GGT participants were invited to continue to exercise at home. IHT participants continued to exercise at home as usual. Adherence was recorded via logs and measured as the percentage of exercise sessions actually performed out of the total number of scheduled sessions in three 1-month periods: one before (PRE) and two after (M1 and M2) the beginning of lockdown. Before lockdown, IHT (66.8% ± 26.6) and GGT (76.3% ± 26.6) adherence were similar. During lockdown, IHT participation increased (M1: 81.5% ± 31.0; M2: 88.0% ± 28.3), while that of GGT showed no statistical differences (M1: 79.4% ± 34.2; M2: 80.6% ± 36.4). Exercise protocols based on supervised gym practice must consider the possibility of disruptive events, which could cause a sudden interruption of gym activity and include educational initiatives to instruct participants to exercise effectively and safely without a trainer’s direct supervision.

## 1. Introduction

Primary osteoporosis (OP) is an age-related systemic disease of the skeleton characterized by a reduced mass and deterioration of the micro-architecture of the bone, accompanied by an increased risk of fracture with consequent pain, decreased physical and social functional capacity, and quality of life (QoL) [1,2,3,4,5,6,7,8,9]. There is a general consensus on the efficacy of physical activity in the prevention of OP and its consequences [10,11]. A meta-analysis, which included 59 randomized controlled trials, demonstrated the efficacy of exercise programs compared to no exercise, sham programs, or pharmacological interventions in women with OP [12]. In the studies included in this meta-analysis, the exercise programs were administered either as individual home training (IHT) or in gym group training (GGT). In trials based on IHT, participants, after an appropriate number of educational sessions, performed exercise individually at home, without the direct supervision of a trainer, delivered to them by an exercise professional in gyms, hospitals, or community health facilities. Moreover, participants were given printed or web-based educational materials to help them in performing the requested exercises in a correct and safe way, autonomously. The time of each exercise session was chosen by the participants when they found it most convenient for them. Conversely, in the GGT, both educational activities and exercises were carried out in gym group sessions with the supervision of the trainer. The number of exercise sessions, their time, and duration were scheduled by the trainer.

Adherence to an exercise program is of fundamental importance for the programs’ efficacy and is problematic in all age groups but particularly among older adults [13,14,15]. Adherence depends on several factors, including personal-level factors and program characteristics [16]. Several personal-level factors, such as poor health, low self-confidence, low motivation, and poor enjoyment of the perceived exercise have been found to be associated with lower adherence [15,17]. Interestingly, reduced mental wellbeing was found to be a greater barrier to exercise adherence than reduced physical wellbeing [16]. Program characteristics also play an important role. Adherence was generally found to be higher in supervised programs than in those unsupervised, as corroborated by systematic reviews [13,16,18,19,20]. GGT could facilitate participation by enhancing social interactions, which lead to improve social, mental, and emotional health [13,16]. In our previous study [15], the adherence to a specifically designed exercise program was found to be the key predictor of improved back pain. Adherence, in turn, was independently associated with accessibility to gyms (shorter home–gym distance) and positive relationship with the trainer [14]. However, regular participation in GGT classes requires compliance with a fixed time schedule, which may not be compatible with family or work needs. On the other hand, IHT participants need to be well-motivated and accurately instructed to exercise autonomously.

To our knowledge, no studies compared the effects of a specific exercise program for women with postmenopausal OP when administered as IHT or GGT. Thus, our study was originally designed to consider whether IHT could be a valid alternative to GGT, since it could overcome problems related to accessibility to gyms or time schedule rigidity. With these premises, within the European project ACTLIFE, we started a randomized trial [21] to verify the efficacy of a physical activity program designed to improve the quality of life in sedentary women with postmenopausal OP, when administered IHT or GGT. During the trial, the COVID-19 pandemic resulted in a government-imposed national lockdown from 9 March–May 18 2020, restricting the movement of the population except for necessity, work, and health circumstances. The lockdown imposed the temporary closure of nonessential businesses, including gym activity, causing the interruption of the GGT but not the IHT.

This study is aimed at investigating how COVID-19 lockdown modified adherence to training practice in the two groups of ACTLIFE project’s participants. We expected the adherence to exercise program of both groups to be severely disrupted during the pandemic. In addition, we hypothesized that IHT participants would be less affected by lockdown restrictions, since they were already instructed to organize their training practice autonomously at home. On the contrary, the GGT participants would have been affected to a greater extent, since they had to reorganize their weekly exercise routine and perform this individually.

## 2. Materials and Methods

When the pandemic erupted and the lockdown was imposed, a randomized controlled study was being conducted. It was aimed at investigating the efficacy of an exercise program for women with primary postmenopausal OP (T score ≤ 2.5), when administered as either GGT or IHT. The study was conducted within the project “Physical ACTivity: the tool to improve the quality of LIFE in osteoporosis people” (ACTLIFE), funded by the European Commission within the Erasmus+ Sport program (Grant Agreement N2017-2128/001-001). The study was conducted according to the guidelines of the Declaration of Helsinki, and approved by the Local Ethics Committee (*Comitato Etico Indipendente di Area Vasta Emilia Centro*) of the Emilia–Romagna Region (reference number AVEC: EM601-2019_696/2018/Sper/IOR_EM2). The trial was registered in ClinicalTrial.Gov (NCT04179903).

Details on study methods have been published previously [21]. Briefly, postmenopausal women with OP were recruited by the *Centro Osteoporosi e Malattie Metaboliche dello Scheletro* of Rizzoli Orthopaedic Institute of Bologna, Italy, and had no significant comorbidities affecting motor or cognitive functions. The exercise program was designed to improve quality of life in the OP population by drawing on the most recent evidence in the sector [22,23,24], with the aim of increasing joint mobility, muscular force, static and dynamic balance, motor coordination, and endurance. The study was a randomized trial with two parallel groups who exercised as IHT or GGT. Each group was scheduled to perform the ACTLIFE physical activity program for 12 months using simple equipment (i.e., mats, sticks, soft balls, elastic bands, weights) in two 1-h sessions per week. Moreover, all participants were requested to choose an additional third day of the week to perform brisk walking, cycling, or swimming for at least 30 min, in order to reach the weekly amount of at least 150 min of exercise recommended by WHO [5]. It was a single-blinded study, since professionals who evaluated the women were not aware of to which exercise group they were assigned. Informed consent was obtained from all subjects involved in the study.

GGT was performed in two 1-h exercise sessions per week in well-equipped gyms under the direct supervision of a graduate trainer. For the IHT group, the trainer explained to the participants how to perform the physical activity at home in two 1-h unsupervised sessions per week. Participants were also given educational material with the purpose of explaining how to correctly perform the exercises. Participants were requested to strictly adhere to the instructions provided. Subsequently, the trainer contacted the IHT participants at pre-established time intervals to encourage them to exercise regularly and to obtain information on their health status. Every 6–8 weeks, a face-to-face appointment was scheduled to review and upgrade the exercise program [21], based on the progression principle.

The study protocol scheduled participants’ evaluations at baseline and after 6 and 12 months. At the baseline, all participants underwent a multidimensional assessment, which included age, body mass index, functional capacity (Short Physical Performance Battery [25,26]), fear of falling (Short Fall Efficacy Scale–International [27,28]), and OP-related quality of life (Assessment of Health-Related Quality of Life in Osteoporosis [29,30]).

Immediately after the beginning of the lockdown, IHT participants were invited to continue their weekly practice as instructed. Those in the GGT group were asked to perform the exercises learned during gym classes at home, with the support of educational material that was sent them via e-mail. For both groups, the trainers kept in touch with all participants, providing instructions and advice by telephone or video calls. Specifically, the participants were instructed to exercise in two 1-h sessions per week.

Participants were requested to record the execution of each exercise session on specific weekly logs, which had been given them by research team. Logs were returned after the end of lockdown. Adherence was measured as the percentage of exercise sessions actually performed out of the total number of planned exercise sessions in three 1-month periods: one before (PRE) and two after (M1 and M2) the date of the beginning of lockdown.

### 2.1. Participants’ Characteristics

When the lockdown was imposed by the Italian national government, 48 postmenopausal women with OP had been participating in the study, but 5 did not fill or return their weekly logs (two in IHT and three in GGT group). Therefore, we included 23 women of the IHT group and 20 of the GGT group in these findings. The time interval from the beginning of the ACTLIFE exercise program and that of the lockdown was 6.8 ± 1.5 (range 5.0–9.6) months for the IHT group and 6.6 ± 1.6 (range 5.0–8.4) months for the GGT group (Student *t*-test *p* > 0.05). No statistically significant difference was observed between the two groups at baseline assessment (Table 1). Only 49% of participants had completed the first 6 months of the study at the beginning of the lockdown.

### 2.2. Statistical Analysis

The Shapiro–Wilk test was used to check the normal distribution of the data. Normal distributed continuous variables were presented as mean and standard deviation (SD), while non-normal distributed variables were presented as median and interquartile range (IQR). Categorical variables were presented as a percentage over the total. The repeated measure ANOVA test was performed to assess the between-group differences of continuous variables, along with the two times assessment, while the two-tailed Student’s *t*-test was used to compare each group with one another. The Mann–Whitney U test was used to compare two groups in case of non-normally distributed variables. Differences between the groups were considered statistically significant if *p* < 0.05. *p*-values were adjusted using the Bonferroni post hoc correction for multiple comparisons. A post hoc power analysis was conducted in G*Power 3.1.9.4 (Franz Paul, Kiel, Germany) to ensure the statistical effectiveness of the results obtained. A minimum power of 0.82 was ensured, accounting for a type I error of 0.05. All statistical analyses were performed in JASP (JASP Team, 2020, Version 0.14.1 (Computer software)).

## 3. Results

**Figure 1:**
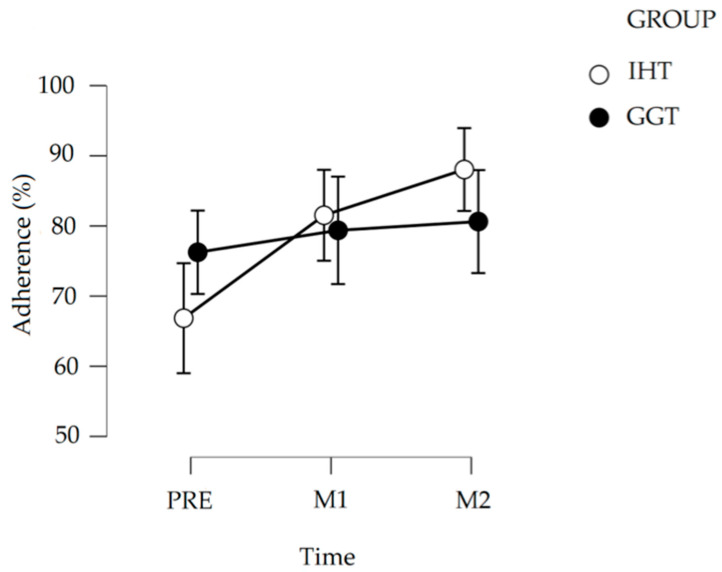
Average percentage of adherence in a three-month period: before (PRE) and one (M1) and two (M2) months after the beginning of lockdown (8 March 2020). The error bars represent the SD.

As shown in Figure 1 and Table 2, adherence before lockdown (PRE) did not differ between the two groups. On the other hand, as shown in Table 3, IHT adherence showed a statistically significant progressive increase from PRE (66.8 ± 37.6%) to M1 (81.5 ± 31.0%) and to M2 (88.0 ± 28.3%). Conversely, in the GGT group, adherence did not statistically change from PRE (76.3 ± 26.6%) to M1 (79.4 ± 34.2%) and M2 (80.6 ± 32.8%).

## 4. Discussion

Participation in and adherence to a program is important to the internal validity of a study, but about 50% of people who embark on an exercise program will drop out within six months [31]. Structured or group programs to increase physical activity in older adults have demonstrated high short-term participation rates and good long-term retention rates [31].

The COVID-19 pandemic and associated restrictions may have disrupted the study participants’ routines and motivation, as staying at home can lead to reduced physical activity and sedentary behavior. In addition, the reduction or lack of social bonds that are usually essential to encourage the elderly to exercise may have reduced.

This study was aimed at evaluating the effects of the COVID-19 lockdown on training practice in postmenopausal women with OP who were administered a specific exercise program as either IHT or GGT. We investigated the adherence to the exercise protocol as the primary outcome measure since, as stated above, it is of fundamental importance to prove its efficacy and validate the expected results [13,14,15]. In general, attending an exercise training program regularly is a challenge, as several factors can serve as barriers to exercise adherence [15,16,32]. In women with postmenopausal OP, adherence to an exercise protocol may also be prevented by fear of falls and fracture [33]. In this scenario of expected low participation, we expected the adherence to drop even more during lockdown, given the great uncertainty caused by the COVID-19 pandemic [34,35,36]. On the contrary, we observed that adherence of both groups at least maintained the levels recorded before the lockdown. With present methods, we cannot empirically prove the reason for this observation. However, we speculate that this result might be due to the educational activity performed within the study to promote an active lifestyle among participants and/or to the increased free time compared to the normal daily routines without lockdown. The ongoing relationship between participants and trainer during lockdown via telephone and social media may have facilitated adherence, as suggested by previous studies [37,38,39,40].

The present study also hypothesized that IHT adherence would have been less affected by lockdown restrictions than that of the GGT, since participants were already instructed to organize their training practice autonomously at home. Indeed, we found a marked increase in weekly training practice in the initial lockdown months in IHT but not in the GGT group. This result endorses the notion of the importance of specific educational strategies to promote a more active lifestyle oriented to the prevention of chronic diseases and their consequences [41].

## 5. Conclusions

Results lead us to conclude that exercise protocols, even if based on supervised gym practice, must consider the possibility that a disruptive event (or, more simply, a change in a person’s daily routine) could cause a sudden interruption of gym participation. Therefore, it is very important to include in the exercise protocols educational approaches to instruct participants to exercise effectively and safely without the direct supervision of a trainer. This needs to be supported by the ongoing relationship and supervision of trainers, which may be facilitated by telephone or other appropriate technological tools [37,38,39,40].

## Figures and Tables

**Table 1:** Characteristics of the IHT and GGT groups at the baseline assessment and adherence before the lockdown.

	IHT Group (N = 23)	GGT Group (N = 20)	Test	*p* *
	Mean (±SD)	Mean (±SD)		
Age (years)	65.6 (±5.6)	65.0 (±7.4)	Student *t*	NS
Body Mass Index	23.5 (±3.1)	23.6 (±4.1)	Student *t*	NS
	Median (IQR)	Median (IQR)		
Short Physical Performance Battery	11.0 (10.0–12.0)	11.0 (9.0–12.0)	Mann–Whitney	NS
Short Fall Efficacy Scale–International	8.0 (7.0–9.0)	8.0 (7.0–9.2)	Mann–Whitney	NS
Assessment of Health-Related Quality of Life in Osteoporosis	1.6 (1.3–1.8)	1.6 (1.3–2.1)	Mann––Whitney	NS

Note: GGT: group gym training group; IHT: individual home training group; SD: standard deviation; IQR: interquartile range; * NS: *p* > 0.05.

**Table 2:** Repeated measure ANOVA.

	F	*p*	η^2^ *_p_*
TIME	13.781	<0.001	0.252
TIME × GROUP	5.936	0.015	0.126

Note: Type III Sum of Squares.

**Table 3:** Post hoc comparison–GROUP * TIME.

		Mean Difference	*p*_bonf_
IHT, PRE	GGT, PRE	−9.402	NS
	IHT, M1	−14.674	<0.001
	GGT, M1	−12.527	NS
	IHT, M2	−21.196	<0.001
	GGT, M2	−13.777	NS
GGT, PRE	IHT, M1	−5.272	NS
	GGT, M1	−3.125	NS
	IHT, M2	−11.793	NS
	GGT, M2	−4.375	NS
IHT, M1	GGT, M1	2.147	NS
	IHT, M2	−6.522	NS
	GGT, M2	0.897	NS
GGT, M1	IHT, M2	−8.668	NS
	GGT, M2	−1.250	NS
IHT, M2	GGT, M2	7.418	NS

Note: *p*-value adjusted through Bonferroni correction; GGT: group gym training group; IHT: individual home training group; before (PRE) and one (M1) and two (M2) months after the beginning of lockdown (8 March 2020).

## Data Availability

The data presented in this study are available on request from the corresponding author. The data are not publicly available due to ethical and privacy reasons.
